# Clinical characteristics and prognostic factors in intracranial hemorrhage patients with hematological diseases

**DOI:** 10.1007/s00277-022-04982-w

**Published:** 2022-09-30

**Authors:** Jia-Yuan Zhang, Ying Li, Yue-Shen Ma, Xiu-Juan Sun, Yong-Ze Liu, Yan-Ke Yin, Bo Hu, Ming-Huan Su, Qiu-Ling Li, Ying-Chang Mi, Da-Peng Li

**Affiliations:** 1 Department of Emergency, State Key Laboratory of Experimental Hematology, National Clinical Research Center for Blood Diseases, Haihe Laboratory of Cell Ecosystem, Institute of Hematology & Blood Diseases Hospital, Chinese Academy of Medical Sciences & Peking Union Medical College, Tianjin, 300020 China; 2 Department of Radiology, State Key Laboratory of Experimental Hematology, National Clinical Research Center for Blood Diseases, Haihe Laboratory of Cell Ecosystem, Institute of Hematology & Blood Diseases Hospital, Chinese Academy of Medical Sciences & Peking Union Medical College, Tianjin, 300020 China; 3 Department of Biostatistics, State Key Laboratory of Experimental Hematology, National Clinical Research Center for Blood Diseases, Haihe Laboratory of Cell Ecosystem, Institute of Hematology & Blood Diseases Hospital, Chinese Academy of Medical Sciences & Peking Union Medical College, Tianjin, 300020 China

**Keywords:** Clinical characteristics, Hematological diseases, Intracranial hemorrhage, Prognosis

## Abstract

The clinical characteristics and prognosis of intracranial hemorrhage (ICH) in patients with hematological diseases remain controversial. This study aimed to describe the clinical characteristics and explore the prognostic factors in such patients. A total of 238 ICH patients with a hematological disease were recruited from the Institute of Hematology and Blood Diseases Hospital, China, from January 2015 to April 2020. The Cox proportional hazards model was used to identify the prognostic factors for 30-day mortality in ICH patients with a hematological disease. There were 123 cases of acute leukemia (AL), 20 of myelodysplasia/myeloproliferative neoplasm, 35 of aplastic anemia (AA), 29 of immune thrombocytopenia (ITP), 19 of congenital/acquired coagulation factor deficiency, and 12 of other hematological diseases. Furthermore, 121 patients presented with a multi-site hemorrhage (MSH), 58 with a single-site hemorrhage in the brain parenchyma (PCH), 23 with a subarachnoid hemorrhage, 33 with a subdural hemorrhage (SH), and three with an epidural hemorrhage. The Cox proportional hazards model indicated association of SH (vs PCH, hazard ratio [HR]: 0.230; 95% confidence interval [CI]: 0.053–0.996; *P* = 0.049), low white blood cells (≤ 100 × 10^9^/L vs > 100 × 10^9^/L, HR: 0.56; 95% CI: 0.348–0.910; *P* = 0.019), AA (vs AL, HR: 0.408; 95% CI: 0.203–0.821; *P* = 0.012), and ITP (vs AL, HR: 0.197; 95% CI: 0.061–0.640; *P* = 0.007) with improved 30-day mortality. However, increased age (HR: 1.012; 95% CI: 1.001–1.022; *P* = 0.034), MSH (vs PCH, HR: 1.891; 95% CI: 1.147–3.117; *P* = 0.012), and a disturbance of consciousness (HR: 1.989; 95% CI: 1.269–3.117; *P* = 0.003) were associated with increased risk of 30-day mortality. In conclusion, in this study, we revealed the clinical characteristics of Chinese ICH patients with a hematological disease. Moreover, we identified risk factors (age, white blood cells, AA, ITP, SH, MSH, and a disturbance of consciousness) that may influence 30-day mortality.

## Introduction

Hematological diseases often present with cerebrovascular complications, including ischemic stroke, intracranial hemorrhage (ICH), microbleeds, posterior reversible encephalopathy syndrome, and dural sinus and cerebral vein thrombosis [1]. A previous study found that the prevalence of stroke in patients with hematological diseases ranged from 0 to 7% [2]. Various hematological disorders may play a direct or indirect role in neurological complications and ICH [3, 4]. Previous studies have already identified hypertension, vessel wall abnormality, invasion or compression of vessels by a tumor in or adjacent to the brain, low platelet count or platelet dysfunction, coagulation factor deficiency, disseminated intravascular coagulation, sepsis, and hyperleukocytosis as independent risk factors for ICH in cancer patients [5–7]. However, the clinical characteristics and prognostic factors in ICH patients with a hematological disease remain controversial.

The pathogenesis of ICH differs in the general and hematological disease populations. The pathogenic mechanisms include abnormal platelet counts or function, coagulation disorders, hyperleukemia, sepsis, and abnormal vessel walls [6–8]. ICH may occur at various timepoints after the diagnosis of hematological disease, especially for patients who firstly experienced hematological disease [9, 10]. Moreover, most hemorrhages in patients with a hematological disease occur in the brain parenchyma, with variable clinical characteristics [11, 12]. The prognosis of ICH patients with hematological diseases has improved because of the development of modern neuroimaging and advances in hematology and neurology [12, 13].

However, epidemiological data on ICH patients with hematological diseases in China are lacking. Therefore, we performed a retrospective single-center study to describe the clinical characteristics and prognostic factors in ICH patients with a hematological disease in China.

## Methods

### Patient population

A total of 238 imaging-confirmed ICH patients with hematological disease attending the Institute of Hematology and Blood Diseases Hospital, Tianjin, China, from January 2015 to April 2020, were analyzed retrospectively. The study was approved by the Institutional Review Board of the Institute of Hematology and Blood Diseases Hospital.

The types of hematological diseases included acute lymphoblastic leukemia, acute myeloid leukemia (AML), acute mixed phenotype leukemia, myelodysplastic syndrome (MDS), atypical chronic granulocytic leukemia (aCML), chronic myelocytic leukemia (CML), chronic myelomonocytic leukemia (CMML), aplastic anemia (AA), immune thrombocytopenia (ITP), hemophilia, acquired hemophilia, vitamin-dependent coagulation factor deficiency, hereditary coagulation factor VII deficiency, non-Hodgkin’s lymphoma (NHL), multiple myeloma (MM), pure red blood cell aplastic anemia (PRCA), Evans syndrome, Fanconi anemia, connective tissue disease combined with abnormal blood cell counts, and hypersplenism. These were grouped into three: Firstly, the MDS/MPN group included MDS, aCML, CML, and CMML. Secondly, the congenital/acquired coagulation factor deficiency (CCFD/ACFD) group included hemophilia, acquired hemophilia, vitamin-dependent coagulation factor deficiency, hereditary coagulation factor VII hemophilia, acquired hemophilia, vitamin-dependent coagulation factor deficiency, and hereditary coagulation factor deficiency. Thirdly, the other group included NHL, MM, PRCA, Evans syndrome, Fanconi anemia, connective tissue disease with abnormal blood counts, and hypersplenism.

Cranial computerized tomography was performed in all patients with a hematological disease, and their cranial hemorrhage site; hemorrhage characteristics, signs, and symptoms; and the presence of any co-infections were recorded. However, the extent of hemorrhage was not known, because most of the hemorrhages were diffuse and multifocal.

ICH included parenchymal hemorrhage (PCH), subarachnoid hemorrhage (SCH), subdural hemorrhage (SH), epidural hemorrhage (EH), and multi-site hemorrhage (MSH). PCH was defined as a single-site hemorrhage that occurred in the brain parenchyma, while MSH was defined as hemorrhages in multiple parts of the brain parenchyma combined with hemorrhages in other parts of the brain parenchyma.

The signs and symptoms included headache, disturbance of consciousness, mental disorder, meningeal irritation sign, hemiplegia, epilepsy, aphasia, and dysopia.

### Data collection and definition

The baseline characteristics recorded included age, sex, hypertension, diabetes mellitus (DM), intracranial hemangioma, and cranial trauma. Patients with acute promyelocytic leukemia (APL) received plasma or fibrinogen infusions if fibrinogen ≤ 1.5 g/L, while platelets were transfused if platelet ≤ 30 × 10^9^/L in these patients. For non-APL patients, red blood cells were infused when hemoglobin ≤ 70 g/L, plasma or fibrinogen was infused if fibrinogen ≤ 1.0 g/L, and platelets were transfused if platelets ≤ 10 × 10^9^/L. Thrombocytopenia was defined as platelets ≤ 30 × 10^9^/L in patients with APL, platelets ≤ 20 × 10^9^/L in patients with acute leukemia other than APL, and platelets ≤ 10 × 10^9^/L in patients with diseases other than these two categories. Coagulation abnormalities were defined as international normalized ratio (INR) > 1.4, and/or partial thromboplastin time (APTT) > 50 s, and/or fibrinogen concentration ≤ 1.5 g/L. Leukocytosis was defined as leukocytes > 100 × 10^9^/L. Serum urea and creatinine levels were also recorded, and patients were accordingly divided into normal and abnormal groups. All data were collected within 3 days for hematological disease patients diagnosed with ICH.

### ICH management and prognosis

Medical management of ICH in patients with a hematological disease included conservative and surgical treatments. Conservative treatment strategies included red blood cell, platelet, plasma, and fibrinogen support therapy; the use of hemostasis and dehydration to reduce cranial pressure; and control of the original disease as well as comorbidities. Surgical treatment was determined by the neurosurgeon after consultation and evaluation of the patient’s condition. Mortality events and the times of mortality were recorded after follow-up to 30 days from the inception of ICH.

### Statistical analysis

Continuous variables are presented as means (standard deviations) or medians (quartiles), according to the data distribution, and categorical variables are presented as numbers (percentages). The differences among groups were calculated using analysis of variance and chi-squared tests. The Kaplan–Meier method was used for survival analysis. Univariate analysis was performed using the Log-rank approach. A Cox proportional hazards model was used to identify the prognostic factors for mortality in ICH patients with a hematological disease. All reported *P* values were two-sided, and statistical significance was set at *P* < 0.05. All statistical analyses were conducted using SPSS software (Version: 22.0; Chicago, IL, USA).

## Results

### Patients’ characteristics

Of the 238 included patients, 123 patients had AL, 20 had MDS/MPN, 35 had AA, 29 had ITP, 19 had CCFD/ACFD, and 12 had other types of hematological diseases. ICH occurred during induction therapy in 218 patients, while 20 patients presented ICH during consolidation therapy. With respect to the bleeding sites, 121 patients presented with MSH, 58 with PCH, 23 with SCH, 33 with SH, and three with EH. We noted that the bleeding sites were different in patients with different types of hematological diseases (*P* < 0.001; Table 1).

**Table 1:** The characteristics of recruited patients

Groups or units	AL (*n* = 123)	MDS/MPN (*n* = 20)	AA (*n* = 35)	ITP (*n* = 29)	CCFD/ACFD (*n* = 19)	Other (*n* = 12)	*P* value
PCH	35 (28.46)	1 (5.00)	6 (17.14)	8 (27.59)	2 (10.53)	6 (50.00)	0.001
SCH	8 (6.50)	5 (25.00)	4 (11.43)	3 (10.34)	3 (15.79)	0 (0.00)	
SH	9 (7.32)	3 (15.00)	3 (8.57)	10 (34.48)	6 (31.58)	2 (16.67)	
EH	1 (0.81)	1 (5.00)	1 (2.86)	0 (0.00)	0 (0.00)	0 (0.00)	
MSH	70 (56.91)	10 (50.00)	21 (60.00)	8 (27.59)	8 (42.11)	4 (33.33)	

*AA*, aplastic anemia; *ACFD*, acquired coagulation factor deficiency; *AL*, acute leukemia; *CCFD*, congenital coagulation factor deficiency; *EH*, epidural hemorrhage; *ITP*, immune thrombocytopenia; *MDS*, myelodysplastic syndrome; *MPN*, myeloproliferative neoplasms; *MSH*, multi-site hemorrhage; *PCH*, parenchyma; *SCH*, subarachnoid hemorrhage; *SH*, subdural hemorrhage

### Clinical characteristics according to the type of hematological disease

The characteristics of recruited patients according to the types of hematological diseases are presented in Table 2. Age (*P* = 0.036), sex (*P* = 0.002), thrombocytopenia (*P* < 0.001), INR (*P* = 0.011), APTT (*P* < 0.001), FIB (*P* < 0.001), white blood cell count (*P* < 0.001), intracranial hemangioma (*P* = 0.006), cranial trauma (*P* = 0.004), disease status (*P* < 0.001), coagulation abnormalities (*P* < 0.001), co-infection (*P* < 0.001), and serum urea (*P* = 0.026) differed significantly among the patients with different types of hematological diseases. However, the distribution of hypertension, diabetes, headache, disturbance of consciousness, mental disorder, meningeal irritation sign, hemiplegia, epilepsy, aphasia, dysopia, and serum creatinine levels were not significantly different among patients with the different types of hematological diseases.

**Table 2:** The clinical characteristics of patients according to the type of hematological disease

Variables	Groups or units	AL (*n* = 123)	MDS/MPN (*n* = 20)	AA (*n* = 35)	ITP (*n* = 29)	CCFD/ACFD (*n* = 19)	Other (*n* = 12)	*P* value
Age (years)	Mean (SD)	36.71 (19.30)	48.50 (17.01)	35.71 (18.43)	42.45 (18.03)	29.51 (24.72)	40.92 (23.18)	0.036
Sex	Male	66 (53.66)	13 (65.00)	19 (54.29)	10 (34.48)	18 (94.74)	5 (41.67)	0.002
	Female	57 (46.34)	7 (35.00)	16 (45.71)	19 (65.52)	1 (5.26)	7 (58.33)	
Thrombocytopenia	Yes	109 (88.62)	17 (85.00)	35 (100.00)	29 (100.00)	1 (5.26)	8 (66.67)	< 0.001
	No	14 (11.38)	3 (15.00)	0 (0.00)	0 (0.00)	18 (94.74)	4 (33.33)	
INR	> 1.4	24 (19.51)	2 (10.00)	0 (0.00)	1 (3.45)	2 (10.53)	0 (0.00)	0.011
	≤ 1.4	99 (80.49)	18 (90.00)	35 (100.00)	28 (96.55)	17 (89.47)	12 (100.00)	
APTT	> 50	4 (3.25)	2 (10.00)	0 (0.00)	1 (3.45)	17 (89.47)	0 (0.00)	< 0.001
	≤ 50	119 (96.75)	18 (90.00)	35 (100.00)	28 (96.55)	2 (10.53)	12 (100.00)	
FIB	> 1.5	78 (63.41)	18 (90.00)	33 (94.29)	26 (89.66)	18 (94.74)	10 (83.33)	< 0.001
	≤ 1.5	45 (36.59)	2 (10.00)	2 (5.71)	3 (10.34)	1 (5.26)	2 (16.67)	
White blood cells*10^9^/L	> 100	37 (30.08)	3 (15.00)	0 (0.00)	0 (0.00)	0 (0.00)	0 (0.00)	< 0.001
	≤ 100	86 (69.92)	17 (85.00)	35 (100.00)	29 (100.00)	19 (100.00)	12 (100.00)	
Hypertension	No	109 (88.62)	17 (85.00)	34 (97.14)	26 (89.66)	17 (89.47)	9 (75.00)	0.399
	Yes	14 (11.38)	3 (15.00)	1 (2.86)	3 (10.34)	2 (10.53)	3 (25.00)	
Diabetes	No	119 (96.75)	17 (85.00)	35 (100.00)	26 (89.66)	19 (100.00)	11 (91.67)	0.062
	Yes	4 (3.25)	3 (15.00)	0 (0.00)	3 (10.34)	0 (0.00)	1 (8.33)	
Intracranial hemangioma	No	119 (96.75)	20 (100.00)	34 (97.14)	29 (100.00)	15 (78.95)	11 (91.67)	0.006
	Yes	4 (3.25)	0 (0.00)	1 (2.86)	0 (0.00)	4 (21.05)	1 (8.33)	
Cranial trauma	No	121 (98.37)	19 (95.00)	34 (97.14)	26 (89.66)	15 (78.95)	12 (100.00)	0.004
	Yes	2 (1.63)	1 (5.00)	1 (2.86)	3 (10.34)	4 (21.05)	0 (0.00)	
Disease status	M3	36 (29.27)	0 (0.00)	0 (0.00)	0 (0.00)	0 (0.00)	0 (0.00)	< 0.001
	Non-M3	87 (70.73)	20 (100.00)	35 (100.00)	29 (100.00)	19 (100.00)	12 (100.00)	
Headache	No	40 (32.52)	4 (20.00)	3 (8.57)	7 (24.14)	6 (31.58)	5 (41.67)	0.077
	Yes	83 (67.48)	16 (80.00)	32 (91.43)	22 (75.86)	13 (68.42)	7 (58.33)	
Disturbance of consciousness	No	100 (81.30)	15 (75.00)	31 (88.57)	24 (82.76)	15 (78.95)	11 (91.67)	0.757
	Yes	23 (18.70)	5 (25.00)	4 (11.43)	5 (17.24)	4 (21.05)	1 (8.33)	
Mental disorder	No	110 (89.43)	19 (95.00)	30 (85.71)	29 (100.00)	18 (94.74)	11 (91.67)	0.390
	Yes	13 (10.57)	1 (5.00)	5 (14.29)	0 (0.00)	1 (5.26)	1 (8.33)	
Meningeal irritation sign	No	111 (90.24)	18 (90.00)	31 (88.57)	29 (100.00)	17 (89.47)	11 (91.67)	0.650
	Yes	12 (9.76)	2 (10.00)	4 (11.43)	0 (0.00)	2 (10.53)	1 (8.33)	
Hemiplegia	No	85 (69.11)	15 (75.00)	30 (85.71)	23(79.31)	14 (73.68)	8 (66.67)	0.454
	Yes	38 (30.89)	5 (25.00)	5 (14.29)	6 (20.69)	5 (26.32)	4 (33.33)	
Epilepsy	No	115 (93.50)	18 (90.00)	34 (97.14)	29 (100.00)	19 (100.00)	12 (100.00)	0.365
	Yes	8 (6.50)	2 (10.00)	1 (2.86)	0 (0.00)	0 (0.00)	0 (0.00)	
Aphasia	No	117 (95.12)	19 (95.00)	34 (97.14)	29 (100.00)	18 (94.74)	10 (83.33)	0.345
	Yes	6 (4.88)	1 (5.00)	1 (2.86)	0 (0.00)	1 (5.26)	2 (16.67)	
Dysopia	No	116 (94.31)	20 (100.00)	35 (100.00)	29 (100.00)	19 (100.00)	12 (100.00)	0.243
	Yes	7 (5.69)	0 (0.00)	0 (0.00)	0 (0.00)	0 (0.00)	0 (0.00)	
Coagulation abnormalities	No	75 (60.98)	17 (85.00)	33 (94.29)	26 (89.66)	0 (0.00)	10 (83.33)	< 0.001
	Yes	48 (39.02)	3 (15.00)	2 (5.71)	3 (10.34)	19 (100.00)	2 (16.67)	
Co-infection	No	35 (28.46)	11 (55.00)	14 (40.00)	21 (72.41)	18 (94.74)	5 (41.67)	< 0.001
	Yes	88 (71.54)	9 (45.00)	21 (60.00)	8 (27.59)	1 (5.26)	7 (58.33)	
Serum Urea	> 8.2	12 (9.76)	7 (35.00)	4 (11.43)	3 (10.34)	1 (5.26)	3 (25.00)	0.026
	≤ 8.2	111 (90.24)	13 (65.00)	31 (88.57)	26 (89.66)	18 (94.74)	9 (75.00)	
Serum creatinine	≤ 106	118 (95.93)	18 (90.00)	34 (97.14)	29 (100.00)	19 (100.00)	11 (91.67)	0.443
	> 106	5 (4.07)	2 (10.00)	1 (2.86)	0 (0.00)	0 (0.00)	1 (8.33)	

*AA*, aplastic anemia; *ACFD*, acquired coagulation factor deficiency; *AL*, acute leukemia; *APTT*, partial thromboplastin time; *CCFD*, congenital coagulation factor deficiency; *FIB*, fibrinogen; *INR*, international normalized ratio; *ITP*, immune thrombocytopenia; *MDS*, myelodysplastic syndrome; *MPN*, myeloproliferative neoplasms

### Clinical characteristics according to bleeding sites

The characteristics of the recruited patients according to the bleeding sites are presented in Table 3. APTT (*P* = 0.009), FIB (*P* = 0.022), white blood cell count (*P* < 0.001), cranial trauma (*P* < 0.001), disease status (*P* = 0.007), headache (*P* = 0.006), disturbance of consciousness (*P* = 0.002), meningeal irritation sign (*P* < 0.001), hemiplegia (*P* < 0.001), and co-infection (*P* = 0.002) were significantly associated with bleeding sites. However, the distribution of age, sex, thrombocytopenia, INR, hypertension, diabetes, intracranial hemangioma, mental disorder, epilepsy, aphasia, dysopia, coagulation abnormalities, serum urea, and serum creatinine did not show a significant association with any bleeding site.

**Table 3:** The clinical characteristics of patients according to the bleeding sites

Variables	Groups or units	PCH (*n* = 58)	SCH (*n* = 23)	SH (*n* = 33)	EH (*n* = 3)	MSH (*n* = 121)	*P* value
Age (years)	Mean (SD)	40.47 (21.05)	38.65 (19.12)	36.99 (20.24)	9.67 (10.79)	37.45 (19.08)	0.186
Sex	Male	32 (55.17)	12 (52.17)	14 (42.42)	2 (66.67)	71 (58.68)	0.558
	Female	26 (44.83)	11 (47.83)	19 (57.58)	1 (33.33)	50 (41.32)	
Thrombocytopenia	Yes	48 (82.76)	20 (86.96)	25 (75.76)	2 (66.67)	104 (85.95)	0.591
	No	10 (17.24)	3 (13.04)	8 (24.24)	1 (33.33)	17 (14.05)	
INR	> 1.4	9 (15.52)	1 (4.35)	2 (6.06)	0 (0.00)	17 (14.05)	0.424
	≤ 1.4	49 (84.48)	22 (95.65)	31 (93.94)	3 (100.00)	104 (85.95)	
APTT	> 50	1 (1.72)	4 (17.39)	8 (24.24)	0 (0.00)	11 (9.09)	0.009
	≤ 50	57 (98.28)	19 (82.61)	25 (75.76)	3 (100.00)	110 (90.91)	
FIB	> 1.5	37 (63.79)	20 (86.96)	30 (90.91)	3 (100.00)	93 (76.86)	0.022
	≤ 1.5	21 (36.21)	3 (13.04)	3 (9.09)	0 (0.00)	28 (23.14)	
White blood cells*10^9^/L	> 100	9 (15.52)	0 (0.00)	0 (0.00)	0 (0.00)	31 (25.62)	< 0.001
	≤ 100	49 (84.48)	23 (100.00)	33 (100.00)	3 (100.00)	90 (74.38)	
Hypertension	No	47 (81.03)	21 (91.30)	32 (96.97)	3 (100.00)	109 (90.08)	0.162
	Yes	11 (18.97)	2 (8.70)	1 (3.03)	0 (0.00)	12 (9.92)	
Diabetes	No	56 (96.55)	20 (86.96)	32 (96.97)	3 (100.00)	116 (95.87)	0.371
	Yes	2 (3.45)	3 (13.04)	1 (3.03)	0 (0.00)	5 (4.13)	
Intracranial hemangioma	No	54 (93.10)	23 (100.00)	32 (96.97)	3 (100.00)	116 (95.87)	0.682
	Yes	4 (6.90)	0 (0.00)	1 (3.03)	0 (0.00)	5 (4.13)	
Cranial trauma	No	55 (94.83)	22 (95.65)	31 (93.94)	0 (0.00)	119 (98.35)	< 0.001
	Yes	3 (5.17)	1 (4.35)	2 (6.06)	3 (100.00)	2 (1.65)	
Disease status	M3	16 (27.59)	2 (8.70)	0 (0.00)	0 (0.00)	18 (14.88)	0.007
	Non-M3	42 (72.41)	21 (91.30)	33 (100.00)	3 (100.00)	103 (85.12)	
Headache	No	23 (39.66)	4 (17.39)	2 (6.06)	0 (0.00)	36 (29.75)	0.006
	Yes	35 (60.34)	19 (82.61)	31 (93.94)	3 (100.00)	85 (70.25)	
Disturbance of consciousness	No	49 (84.48)	23 (100.00)	32 (96.97)	3 (100.00)	89 (73.55)	0.002
	Yes	9 (15.52)	0 (0.00)	1 (3.03)	0 (0.00)	32 (26.45)	
Mental disorder	No	49 (84.48)	22 (95.65)	32 (96.97)	3 (100.00)	111 (91.74)	0.240
	Yes	9 (15.52)	1 (4.35)	1 (3.03)	0 (0.00)	10 (8.26)	
Meningeal irritation sign	No	54 (93.10)	15 (65.22)	32 (96.97)	3 (100.00)	113 (93.39)	< 0.001
	Yes	4 (6.90)	8 (34.78)	1 (3.03)	0 (0.00)	8 (6.61)	
Hemiplegia	No	43 (74.14)	22 (95.65)	32 (96.97)	3 (100.00)	75 (61.98)	< 0.001
	Yes	15 (25.86)	1 (4.35)	1 (3.03)	0 (0.00)	46 (38.02)	
Epilepsy	No	54 (93.10)	20 (86.96)	33 (100.00)	3 (100.00)	117 (96.69)	0.160
	Yes	4 (6.90)	3 (13.04)	0 (0.00)	0 (0.00)	4 (3.31)	
Aphasia	No	53 (91.38)	23 (100.00)	33 (100.00)	3 (100.00)	115 (95.04)	0.290
	Yes	5 (8.62)	0 (0.00)	0 (0.00)	0 (0.00)	6 (4.96)	
Dysopia	No	55 (94.83)	23 (100.00)	33 (100.00)	3 (100.00)	117 (96.69)	0.584
	Yes	3 (5.17)	0 (0.00)	0 (0.00)	0 (0.00)	4 (3.31)	
Coagulation abnormalities	No	35 (60.34)	16 (69.57)	23 (69.70)	3 (100.00)	84 (69.42)	0.539
	Yes	23 (39.66)	7 (30.43)	10 (30.30)	0 (0.00)	37 (30.58)	
Co-infection	No	16 (27.59)	13 (56.52)	21 (63.64)	3 (100.00)	51 (42.15)	0.002
	Yes	42 (72.41)	10 (43.48)	12 (36.36)	0 (0.00)	70 (57.85)	
Serum Urea	> 8.2	7 (12.07)	2 (8.70)	6 (18.18)	0 (0.00)	15 (12.40)	0.791
	≤ 8.2	51 (87.93)	21 (91.30)	27 (81.82)	3 (100.00)	106 (87.60)	
Serum creatinine	≤ 106	57 (98.28)	23 (100.00)	32 (96.97)	3 (100.00)	114 (94.21)	0.546
	> 106	1 (1.72)	0 (0.00)	1 (3.03)	0 (0.00)	7 (5.79)	

*APTT*, partial thromboplastin time; *EH*, epidural hemorrhage; *FIB*, fibrinogen; *INR*, international normalized ratio; *MSH*, multi-site hemorrhage; *PCH*, parenchyma; *SCH*, subarachnoid hemorrhage; *SH*, subdural hemorrhage

### Prognostic factors

The prognostic factors for 30-day mortality in ICH patients with a hematological disease are presented in Table 4. Univariate analysis showed that SCH (hazards ratio [HR]: 0.101; 95% confidence interval [CI]: 0.013–0.809; *P* = 0.031) and SH (HR: 0.187; 95% CI: 0.041–0.858; *P* = 0.031) were associated with an improvement in the 30-day mortality, as compared with PCH. Moreover, patients with M3 hematological disease had better 30-day mortality than those with non-M3 hematological disease (HR: 0.425; 95% CI: 0.209–0.866; *P* = 0.018). Furthermore, patients with AA (HR: 0.294; 95% CI: 0.135–0.639; *P* = 0.002) and ITP (HR: 0.135; 95% CI: 0.038–0.477; *P* = 0.002) had a better prognosis than those with AL. Finally, disturbance of consciousness (HR: 2.231; 95% CI: 1.294–3.846; *P* = 0.004) and coagulation abnormalities (HR: 4.823; 95% CI: 1.014–22.935; *P* = 0.048) were associated with a poor prognosis in terms of 30-day mortality.

**Table 4:** The prognostic factors of 30-day mortality

Variable	Univariate analysis				Multivariate analysis			
	*P* value	HR	HR low limit	HR upper limit	*P* value	HR	HR low limit	HR upper limit
Age	0.104	1.012	0.998	1.026	**0.034**	**1.012**	**1.001**	**1.022**
Sex	0.249	1.316	0.825	2.100	-	-	-	-
SCH vs PCH	0.031	0.101	0.013	0.809	0.058	0.140	0.019	1.064
SH vs PCH	0.031	0.187	0.041	0.858	0.049	0.230	0.053	0.996
EH vs PCH	0.987	0.000	0.000		0.985	0.000	0.000	
MSH vs PCH	0.055	1.706	0.989	2.944	0.012	1.891	1.147	3.117
Thrombocytopenia	0.111	0.454	0.171	1.200	-	-	-	-
INR	0.557	1.243	0.601	2.572	-	-	-	-
APTT	0.150	3.157	0.661	15.078	-	-	-	-
FIB	0.250	0.412	0.091	1.864	-	-	-	-
White blood cells	0.156	0.668	0.383	1.166	0.019	0.563	0.348	0.910
Hypertension	0.774	0.881	0.373	2.082	-	-	-	-
Diabetes	0.748	0.801	0.208	3.086	-	-	-	-
Intracranial hemangioma	0.282	0.459	0.111	1.900	-	-	-	-
Cranial trauma	0.622	0.584	0.069	4.944	-	-	-	-
M3 vs non-M3	0.018	0.425	0.209	0.866	-	-	-	-
MDS/MPN vs AL	0.607	0.794	0.330	1.910	0.408	0.727	0.342	1.547
AA vs AL	0.002	0.294	0.135	0.639	0.012	0.408	0.203	0.821
ITP vs AL	0.002	0.135	0.038	0.477	0.007	0.197	0.061	0.640
CCFD/ACFD vs AL	0.601	0.611	0.096	3.871	0.181	0.495	0.177	1.386
Other vs AL	0.083	0.275	0.064	1.182	0.093	0.295	0.071	1.227
Headache	0.562	1.184	0.670	2.093	-	-	-	-
Disturbance of consciousness	0.004	2.231	1.294	3.846	**0.003**	**1.989**	**1.269**	**3.117**
Mental disorder	0.836	0.920	0.421	2.014	-	-	-	-
Meningeal irritation sign	0.930	1.042	0.418	2.598	-	-	-	-
Hemiplegia	0.152	1.435	0.875	2.354	-	-	-	-
Epilepsy	0.573	1.426	0.415	4.900	-	-	-	-
Aphasia	0.100	2.110	0.867	5.133	-	-	-	-
Dysopia	0.297	0.521	0.153	1.775	-	-	-	-
Coagulation abnormalities	0.048	4.823	1.014	22.935	-	-	-	-
Co-infection	0.678	0.897	0.536	1.500	-	-	-	-
Serum Urea	0.323	0.687	0.327	1.445	-	-	-	-
Serum creatinine	0.431	1.503	0.545	4.142	-	-	-	-

*AA*, aplastic anemia; *ACFD*, acquired coagulation factor deficiency; *AL*, acute leukemia; *APTT*, partial thromboplastin time; *CCFD*, congenital coagulation factor deficiency; *EH*, epidural hemorrhage; *FIB*, fibrinogen; *HR*, hazards ratio; *INR*, international normalized ratio; *ITP*, immune thrombocytopenia; *MDS*, myelodysplastic syndrome; *MPN*, myeloproliferative neoplasms; *MSH*, multi-site hemorrhage; *PCH*, parenchyma; *SCH*, subarachnoid hemorrhage; *SH*, subdural hemorrhage

[Bold] indicated risk factors

Multivariate analysis showed that SH was associated with a better 30-day mortality (HR: 0.230; 95% CI: 0.053–0.996; *P* = 0.049), while MSH was associated with a poor 30-day mortality, as compared with PCH (HR: 1.891; 95% CI: 1.147–3.117; *P* = 0.012). Moreover, a low white blood cell count was associated with a lower risk of 30-day mortality (HR: 0.563; 95% CI: 0.348–0.910; *P* = 0.019). Furthermore, AA (HR: 0.408; 95% CI: 0.203–0.821; *P* = 0.012) and ITP (HR: 0.197; 95% CI: 0.061–0.640; *P* = 0.007) were associated with an improved 30-day mortality, as compared with AL. Finally, increased age (HR: 1.012; 95% CI: 1.001–1.022; *P* = 0.034) and presentation with a disturbance of consciousness (HR: 1.989; 95% CI: 1.269–3.117; *P* = 0.003) were associated with an increased risk of 30-day mortality.

## Discussion

ICH is more common in patients with a hematological disease than in the general population, and is a serious complication and cause of death in these patients [14–18]. In this study, 238 patients with a hematological disease who presented with ICH were retrospectively recruited and their characteristics observed. Most patients with hematological disease who developed ICH had MSH. The type of hematological disease in patients with ICH differed by age, sex, thrombocytopenia, INR, APTT, FIB, white blood cell count, intracranial hemangioma, cranial trauma, disease status, coagulation abnormalities, co-infection, and serum urea. Furthermore, bleeding sites differed by APTT, FIB, white blood cell count, cranial trauma, disease status, headache, disturbance of consciousness, meningeal irritation sign, hemiplegia, and co-infections in patients with a hematological disease. Finally, the 30-day mortality for ICH patients with hematological disease may be influenced by age, white blood cell count, AA, ITP, SH, MSH, and presentation with a disturbance of consciousness.

This study described the clinical characteristics of ICH in Chinese patients with a hematological disease, which have not been reported previously, and identified prognostic factors for the 30-day mortality in these patients. A previous study conducted by Chen et al. recruited 2574 adult patients with hematological malignancies and found that the incidence of ICH was 2.8% and that the risk of ICH in patients with AML was higher than that in patients with other hematological malignancies [19]. They found that patients who presented with central nervous system–involved lymphomas had a higher risk of ICH than patients with central nervous system–involved AL [19]. Furthermore, the type of hematological malignancies was not associated with mortality [19]. However, that study did not focus on ICH patients with a hematological disease, and the prognostic factors for the 30-day mortality in ICH patients with a hematological disease were not identified. Therefore, we performed this retrospective study to describe the clinical characteristics of ICH patients with a hematological disease and to identify the prognostic factors for 30-day mortality in these patients.

In our study, most patients had MSH; this was not consistent with findings from prior studies [20–22], which found that the most common site of ICH in hematological disorders was a single cortico-parietal area, with much lower incidences of subcortical and cerebellar hemorrhages. Moreover, the most common site of cerebral hemorrhage in patients with hypertension was a hemorrhage in the basal ganglia region. We noted that the sites of ICH varied among the different hematological diseases: (1) The most common bleeding sites for AL patients were MSH and PSH; (2) MSH and SCH were the most common sites in MDS/MPN and AA patients; (3) the most common bleeding site in ITP patients was SH; and (4) MSH and SH were the most common sites in CCFD/ACFD patients. Finally, the symptoms of headache, nausea, vomiting, lateral motor and sensory disturbances, and disturbance of consciousness were relatively common, while mental disorders, meningeal irritation sign, epilepsy, aphasia, and dysopia were relatively uncommon.

Our study found there were significant differences in the age, sex, thrombocytopenia, INR, APTT, FIB, white blood cell counts, intracranial hemangioma, cranial trauma, disease status, coagulation abnormalities, co-infections, and serum urea levels, among patients with the various types of hematological diseases. However, these results were obtained from univariate analysis, and the patients included had various types of hematological diseases, which could have caused some instability in the results. Furthermore, the bleeding sites in patients with a hematological disease may have been affected by APTT, FIB, white blood cell count, cranial trauma, disease status, headache, disturbance of consciousness, meningeal irritation sign, hemiplegia, and co-infection. However, the vascular risk factors did not affect the bleeding sites. These factors included hypertension, diabetes, intracranial hemangioma, and cranial trauma, which were significantly different from the general population and reflect the unique pathophysiological characteristics of ICH in patients with a hematological disease [23–25]. This may have been due to the elevated levels of the white blood cells, resulting from infiltration of the vascular wall, as these patients are prone to vascular endothelial injuries, and release of procoagulant substances and inflammatory markers, which could increase the risk of ICH [26, 27]. Moreover, the APTT and FIB values reflected coagulation abnormalities, which was associated with an increased risk of bleeding [28, 29]. The signs and symptoms of patients may reflect the severity of disease, with the distribution being related significantly to the bleeding sites.

This study found that the 30-day mortality in ICH patients with a hematological disease was 41.2%, which was significantly higher than that in the general population with ICH [15, 30]. The prognostic factors for the 30-day mortality in ICH patients with a hematological disease included age, white blood cell count, AA, ITP, SH, MSH, and a disturbance of consciousness. This may have been because ICH was regarded as secondary to vascular rupturing caused by the leukemia and thrombosis and necrosis of the ischemic tissue [31]. Moreover, AA and ITP are considered to be benign hematological diseases, and prognosis for ICH patients with AA and ITP is relatively better than that of AL patients with ICH, which may be attributed to AL patients being associated with and being more prone to co-infections, coagulation abnormalities, and elevated white blood cell levels. Furthermore, the bleeding sites and the presence of a disturbance of consciousness have been significantly related to the severity of the disease, which may have affected the prognosis of ICH patients with a hematological disease [12]. Finally, infection has been reported to be the main cause of death in patients with hematological malignancies [32]. In our study, most death events (82, 34.5%) occurred within 4 days in patients diagnosed with ICH, while progression of disease or infection contributed less to death events within 4 days.

Certain shortcomings of this study should be acknowledged. First, the current study was a retrospective study, and thus, selective and recall biases were inevitable. Second, the number of patients in the various hematological disease groups was not balanced. Third, the chronic worsening thrombocytopenia and transfusion refractoriness in patients with MDS or CMML resulted in a poor treatment effect and poor prognosis. Fourth, data on the treatment strategies used for hematological diseases, which may have affected the prognosis of ICH patients with a hematological disease, were not available. Finally, stratified analyses were not performed due to the small number of patients with a specific bleeding site.

## Conclusions

The findings of this study systematically describe the clinical characteristics of Chinese ICH patients with a hematological disease. Most of our patients had MSH, and age, sex, thrombocytopenia, INR, APTT, FIB, white blood cells, intracranial hemangioma, cranial trauma, disease status, coagulation abnormalities, co-infection, and serum urea differed among patients with various types of hematological diseases. Moreover, APTT, FIB, white blood cell, cranial trauma, disease status, headache, disturbance of consciousness, meningeal irritation sign, hemiplegia, and co-infection were significantly associated with the bleeding sites. Finally, age, white blood cells, AA, ITP, SH, MSH, and disturbance of consciousness were significantly associated with 30-day mortality in ICH patients with a hematological disease. Further prospective studies should be performed to verify the findings of this study and to assess the prognostic factors in patients with a specific hematological disease.

## Data Availability

All relevant summary data are provided in the manuscript text, and tables. Further inquiries can be directed to the corresponding author.
